# Salt mine microorganisms used for the biotransformation of chlorolactones

**DOI:** 10.1371/journal.pone.0197384

**Published:** 2018-05-17

**Authors:** Wanda Mączka, Małgorzata Grabarczyk, Katarzyna Wińska, Elżbieta Gębarowska, Tomasz Strzała, Marek Durajczyk

**Affiliations:** 1 Department of Chemistry, Faculty of Biotechnology and Food Science, Wrocław University of Environmental and Life Sciences, Wrocław, Poland; 2 Agricultural Microbiology Lab, Department of Plant Protection, Faculty of Life Sciences and Technology, Wrocław University of Environmental and Life Sciences, Wrocław, Poland; 3 Department of Genetics, Faculty of Biology and Animal Science, Wrocław University of Environmental and Life Sciences, Wrocław, Poland; Tallinn University of Technology, ESTONIA

## Abstract

The aim of the project was to find new catalysts capable of chlorolactone biotransformation. Three bicyclic chlorolactones with structures possessing one or two methyl groups in their cyclohexane ring were subjected to screening biotransformation using seven bacterial strains and one fungal strain from a salt mine. Three strains of bacteria (*Micrococcus luteus* Pb10, *Micrococcus luteus* WSP45, *Gordonia alkanivorans* Pd25) and one fungal strain (*Aspergillus sydowii* KGJ10) were able to catalyse hydrolytic dehalogenation of one substrate. The classification of the strains that were effective biocatalysts was confirmed by 16S rDNA analysis. The best result (76%) was obtained using *Aspergillus sydowii* KGJ10. All strains catalysed hydrolytic dehalogenation without changing the conformation. The equatorial position of the chlorine atom in the substrate turned out to be warrant of the positive result of the biotransformation process.

## Introduction

Halogenated organic compounds constitute one of the largest groups of environmental chemicals [1]. Interestingly, a great variety of organohalides are produced naturally. Over 4000 halogenated compounds have been identified to date, in almost all classes of organic chemicals [2]. It has been calculated that 20% of all pharmaceuticals are halogenated [3]. Examples of halogenated natural products include antibiotics (chloramphenicol, 7-chlortetracycline, drosophilin, vancomycin) and anticancer agents such as salinosporamide A from *Salinispora tropica*, the macrocyclic lactone polyether spongistatin, the indolocarbazole rebeccamycin, and the enediyne calicheamicin γ. In turn, neomangicol A and B have demonstrated *in vitro* cytotoxic activity toward the HCT-116 human colon tumor cell line [2]. Another example of a halogen containing drug is metoclopramide, a dopamine receptor antagonist used in the treatment of vomiting and nausea [4]. Many agricultural products such as herbicides, fungicides and insecticides are also halogenated compounds [5,6].

The great utility of halogenated compounds means that it is very important to find effective methods for their degradation, in order to minimise their negative impact on the environment and avoid toxic, carcinogenic, and teratogenic effects on both higher and lower forms of life, including humans [7]. Microorganisms are the major mediators of the cycling of halogenated organic compounds in the environment [8]. The first step in their biodegradation can be dehalogenation, which is catalysed enzymatically [9]. Dehalogenation is also a form of microbiological functionalisation of organic compounds. Filamentous fungi of the genus *Fusarium* have mainly been used for the biotransformation of halogenated compounds until now. These fungi are able to carry out hydrolytic dehalogenation, thereby transforming halolactones into their corresponding hydroxylactones [10–15]. Because halogenated organic compounds can be toxic to microorganisms we decided to search for new biocatalysts among halophiles. Halophilic microbes are capable of degrading organic molecules such as chlorinated compounds [16]. Extreme environments are interesting as sources of microorganisms with exceptional phenotypic and genotypic characteristics [17]. Salt mines can be an effective source of halophiles or halotolerant microbes and in this study were used new cultures of *Micrococcus luteus*, *Gordonia alcanivorans* and *Aspergillus sydowii* from the salt mine KGHM Polska Miedź S.A., Zakłady Górnicze "Polkowice-Sieroszowice".

To our best knowledge there is no previous information in the literature about dehalogenation catalysed by the strains used in the present work.

## Materials and methods

### Chemistry

Progress of reactions and purity of products obtained during syntheses and biotransformation were performed on silica gel-coated aluminium plates (DC-Alufolien Kieselgel 60 F254, Merck, Darmstadt, Germany) with a mixture of hexane and acetone. GC analysis of all compounds was carried out on an Agilent Technologies 6890N instrument (Varian, Agilent Technologies, Santa Clara, CA, USA) using a DB-17 column (cross-linked methyl silicone gum, 30 m × 0.32 mm × 0.25 μm). The enantiomeric excess of the product obtained during biotransformation was determined by GC analysis using the chiral column CP-cyclodextrin-B-225 (30 m × 0.25 mm × 0.25 μm) under the following conditions: injector 200 °C, detector (FID) 250 °C, column temperature: 140 °C (hold 1 min), 140–160 °C (rate 0.5 °C/min), and 160–200 °C (rate 10 °C/min) and 200 °C (hold 1 min).

^1^H NMR spectra were recorded in a CDCl_3_ solution on a Bruker Avance DRX 300 MHz spectrometer (Bruker, Billerica, MA, USA) or on a Bruker Avance^™^ 600 MHz spectrometer (Bruker, Billerica, MA, USA). The molar mass of the new product was confirmed by high resolution mass spectrometry analysis using a Waters LCT Premier XE instrument (ESI ionisation) (Waters Division, Milford, MA, USA). Optical rotation was determined on a P-2000 polarimeter (Jasco Easton, PA, USA) in chloroform solutions.

#### 2-Chloro-5,5-dimethyl-9-oxabicyclo[4.3.0]nonan-8-one (1)

Compound **1** was obtained according to the procedure described earlier [14].^1^H NMR (300 MHz, CDCl_3_, δ, ppm): 0.96 and 1.09 (two s, 6H, CH_3_-9, CH_3_-10), 1.45–1.50 (m, 2H, CH_2_-4), 1.83–1.91 (m, 1H, one of CH_2_-3), 2.00–2.10 (m, 1H, one of CH_2_-3), 2.33 (d, J = 6.8 Hz, 1H, one of CH_2_-7), 2.37 (d, J = 1.7 Hz, 1H, one of CH_2_-7), 2.46–2.50 (m, 1H, H-6), 3.75 (ddd, J = 14.1, 9.0 and 5.1 Hz, 1H, H-2), 4.54 (dd, J = 9.0 and 6.9 Hz, 1H, H-1).

#### 2-Chloro-5-methyl-9-oxabicyclo[4.3.0]nonan-8-one (2)

Compound **2** was obtained according to the procedure described earlier [15].^1^H NMR (600 MHz, CDCl_3_, δ, ppm): 1.01 (d, J = 6.5 Hz, 3H, CH_3_-9), 1.28–1.32 (m, 1H, H-5), 1.52–1.62 (m, 2H, CH_2_-4), 1.98–2.02 (m, 2H, CH_2_-3), 2.23–2.26 (m, 1H, H-6), 2.43 (d, J = 17.0 Hz, 1H, one of CH_2_-7), 2.67 (dd, J = 17.0 and 6.7 Hz,1H, one of CH_2_-7), 4.53 (m, 2H, H-1 and H-2).

#### 2-Chloro-4-methyl-9-oxabicyclo[4.3.0]nonan-8-one (3)

Compound **3** was obtained according to the procedure described earlier [18]. ^1^H NMR (600 MHz, CDCl_3,_ δ, ppm) 0.93 (d, J = 6.5 Hz, 3H, CH_3_-9), 1.61 (ddd, J = 14.8, 11.8 and 3.2 Hz, 2H, CH_2_-5), 1.71–1.79 (m, 1H, one of CH_2_-3), 1.88–1.94 (m, 1H, one of CH_2_-3), 1.98–2.04 (m, 1H, H-4), 2.23 (d, J = 16.4 Hz, 1H, one of CH_2_-7), 2.68 (dd, J = 16.4 and 6.2 Hz, 1H, one of CH_2_-7), 2.69–2.75 (m, 1H, H-6), 4.47–4.49 (m, 1H, H-2), 4.55 (dd, J = 5.5 and 2.7 Hz, 1H, H-1).

### Microorganisms

Seven strains of bacteria: *Aerococcus viridans* WSP451, actinomyces strain Pd25, *Micrococcus luteus* (strains Pb10, Pd31, Pk31, Ob1 WSP45) and one strain of filamentous fungi *Aspergillus* spp. KGJ10 were used in this study. They were isolated from bioaerosols emanating from the salt mine (Salt Mine KGHM Polska Miedź S.A. "Polkowice-Sieroszowice", located in Polkowice, Lower Silesia). *A*. *viridans* WSP451 was isolated from the Vent (the places where the ventilation ducts are connecting and leaving corridors) and other strains from the Working face (the locations where combine mining reaches the solid rock). Air samples were collected from measurement points situated about 400 m underground (air of temperature: 37–39°C, air of humidity: 15–20%) using the impact method and the Air Ideal 3P sampler. For the isolation of bacteria, SNA medium (Standard Nutrient Agar I) containing 6% NaCl was used. In order to eliminate fungi and yeast from the bacteria samples, 30 μg/mL of nystatin was added to the SNA medium. The plates were incubated at 37 °C for 1 day, and at 22 °C for the next 3 days. The bacterial pure cultures were identified according to their morphological and biochemical features (Gram and spore staining as well as catalase and API tests, produced by bioMérieux SA, France, respectively).

For the isolation of fungi, PDA medium (Potato Dextrose Agar, Difco) was used. The samples were incubated at 30 °C for 4 days, and at 22 °C for the next 4 days. The colonies of fungi were identified using the diagnostic keys based on their macroscopic and microscopic morphology [19,20]

### Molecular and data analyses

The selected strains of bacteria (Pd25 and Pb10) and fungi (KGJ10), which were able to perform biotransformation process have been also identified genotypically. DNA was isolated from two bacterial samples (Pd25 and Pb10) and one fungal sample from cultures growing on slants with TSA medium or PDA medium, respectively. A part of the slant was placed in a 1.5 mL tube along with 500 μL of TE buffer. Tubes were then heated in a microwave oven at 600–700 W for two minutes. After heating, the tubes were centrifuged at ~ 6000 g for two minutes and the supernatant from each tube was transferred onto DNAeasy Blood and Tissue kit a spin column (Qiagen, Hilde, Germany). DNA was purified according to the manufacturer’s instructions and finally suspended in PCR grade water.

After isolation, the DNA was used for PCR amplification of the 16S rDNA fragment for bacteria and the internal transcribed fragment (ITS) for fungal samples. Bacterial samples were amplified using primers 27F [21] and 926R [22] and fungal samples were amplified with primers ITS4 and ITS5 [23], both with negative controls run in parallel. The PCR conditions were as follows: initial denaturation 95 °C– 2 min, 35 cycles: 95 °C– 60 s, 50/55* °C– 60 s, 72 °C– 90 s, final elongation 72 °C– 10 min (* 50 °C for 27F/926R; 55 °C for ITS4/ITS5). After amplification, PCR products were checked and quantified using agarose gel electrophoresis and sequenced in both directions using an Applied Biosystems 3730 XL DNA analyzer in Genomed S.A.

Sequences from both strands of each DNA fragment were aligned with Bioedit [24] and a consensus sequence for each sample was created. Next, the systematic position of all sequences were checked in Genbank (http://www.ncbi.nlm.nih.gov) using Blast [25]. For samples where we were not able to recognize a species level systematic position using Blast, phylogenetic analysis was performed using reference homologous sequences from the Biological Resource Center (NRBC—http://www.nite.go.jp) database. Bacterial samples were analysed along with 29 sequences of *Gordonia* species representatives and two samples of *Micrococcus* species used as an outgroup (S1 Table). The fungal sample was analysed along with 147 sequences of *Aspergillus* species representatives and three representatives of *Eupenicillium* sp. used as an outgroup (S2 Table). Trees for both samples were created with Bayesian approach (BA) and a Maximum Likelihood (ML) method. Bayesian trees were estimated with MrBayes 3.2 [26] using the GTR +G +I substitution model (for bacteria) and SYM +G +I (for fungus) as best-fit models selected with jModelTest 2.1.10 [27]. Two independent runs (each with four chains) starting from random trees were applied, trees were sampled every 100^th^ generation for 10,000,000 generations (with 25% burn-in). Analyses were finished, when the average standard deviation of split frequencies was stabilized below 0.01 for all trees used to construct the consensus tree. Maximum likelihood trees were calculated with Phyml [28] online using the newly implemented function of Smart Model Selection function (SMS). We used five random starting trees and SPR type tree improvement. Tree topology was tested by bootstrap analysis using 1000 replications.

### Screening procedure

Biotransformation were performed in two Erlenmeyer flasks of capacity 300 mL with medium containing 2% glucose, 1% peptone, 0.2% casein hydrolysate, 0.2% yeast extract, and 6% NaCl dissolved in 100 mL of distilled water. After three days, when the culture was grown, 10 mg of the substrate dissolved in 1mL of acetone was added to each flask. The shaken cultures were incubated with substrate for the next seven days. After three, five and seven days of incubation in temperature 23 °C, samples containing unreacted substrate, product and mycelium were taken, extracted with dichloromethane (15 mL) and analysed by GC on a DB-17 column after evaporation.

### Preparative biotransformation

In this step 100 mg of substrate **1** dissolved in 10 mL acetone was added to ten Erlenmeyer flasks (300 mL) containing three-day cultures of the fungal strain. After seven days, the entire content of these flasks was extracted with dichloromethane (3 × 40 mL). The combined organic fractions were dried over anhydrous magnesium sulphate and the solvent was evaporated *in vacuo*. In order to separate the pure product from the unreacted substrate and fungal metabolites, column chromatography (silica gel, hexane: acetone 3:1) was performed. The physical and spectral data of **2-hydroxy-5,5-dimethyl-9-oxabicyclo[4.3.0]nonan-8-one** (**4**) are presented below and in S1–S5 Figs:

Isolated yield: 0.0262g (32.1%); (ee = 2.5%, [α]_D_^20^ = +7.22 (c = 0.765g/100mL); ^1^H NMR (600 MHz, CDCl_3_): 0.99 and 1.11 (two s, 6H, CH_3_-9, CH_3_-10), 1.49–1.53 (m, 2H, CH_2_-4), 1.66 (m, 1H, OH), 1.88–1.91 (m, 1H, one of CH_2_-3), 2.08–2.11 (m, 1H, one of CH_2_-3), 2.36–2.40 (m, 2H, CH_2_-7), 2.51–2.55 (m, 1H, H-6), 3.78 (ddd, J = 14.0, 9.0 and 5.0 Hz, 1H, H-2), 4.56–4.59 (dd, J = 9.0 and 7.0 Hz, 1H, H-1), ^13^C NMR (151 MHz, CDCl_3_): 26.95 (C-9), 28.07 (C-10), 29.26 (C-3), 29.76 (C-7), 31.28 (5), 32.75 (C-4), 47.04 (C-6), 59.31 (C-2), 82.96 (C-1), 174.94 (C-8), *ESIHRMS*: calcd for C_10_H_16_O_3_Na, *m*/*z* 207.0997 (M + H)^+^, found 207.0994.

## Results and discussion

During enzymatic dehalogenation of organochlorine compounds, chloride ion is released. We wanted to find chloride-resistant microorganisms, which thus have high tolerance to halogenated compounds. A good source of those microorganisms could be salt mines.

Three bicyclic chlorolactones with the *gem*-dimethylcyclohexane ring (**1**) and the methylcyclohexane ring (**2** and **3**) were used as substrates for biotransformation by microorganisms isolated from bioaerosol emanating from the salt mine (Salt Mine KGHM "Polkowice-Sieroszowice"). The present work is the first report about the use of strains from this environment as biocatalysts. Seven bacterial strains: *Aerococcus viridans* WSP451, *Gordonia alkanivorans* Pd25, *Micrococcus luteus* (strains Pb10, Pd31, Pk31, Ob1, and WSP45), and one strain of a filamentous fungus *Aspergillus sydowii* KGJ10 were used in biotransformation.

It is known from literature that *M*. *luteus* is an ubiquitous gram-positive, strictly aerobic bacterial strain [29]. Several strains of *Micrococcus* sp. have been isolated in previous studies that are able to degrade 2-nitrotoluene, triphenylmethane, nitrobenzene, anthracene, melamine formaldehyde, polyacrylonitrile and azo dyes [30–32]. There are only few reports concerning the use of *M*. *luteus* in biotransformations, which describe the transformation of steroids and oleic acid [33,34].

Another bacteria strain, *G*. *alkanivorans*, was able to reduce ethyl 3-oxo-5-phenylpentanoate [35] and oxidise benzyl alcohol [36].

The only fungal strain used as biocatalyst in the present work, *A*. *sydowii*, was able to catalyse asymmetric bioreduction of 4-methoxyacetophenone [37], hydroxylation of the natural products (–)-ambrox^®^, (–)-sclareol, and (+)-sclareolide [38]; and transformation of 2-phenylacetonitrile to 2-hydroxyphenylacetic acid [39]. This strain can also degrade lignin [40] methyl parathion chlorpyrifos [41,42]–an important organophosphate pesticides, and 1,1-dichloro-2,2-bis-(4-chlorophenyl)ethane (DDD)–a chlorinated aromatic insecticide [43].

In an experiment designed to test the ability of the chosen strains to transform substrates into new and interesting products, screening biotransformations were conducted in the first stage of the study. The results of the screening biotransformations are presented in Table 1.

**Table 1:** Results of the screening biotransformations after three, five and seven days (according to GC).

Entry	Strain	Time [days]	Substrate 1 [%]	Product 4 [%]
**1**	*G*. *alkanivorans* Pd25	3	100	-
		5	100	-
		7	82.2	17.8
**2**	*M*. *luteus* Pb10	3	96.6	3.4
		5	88.3	11.7
		7	76.5	23.5
**3**	*M*. *luteus* Pd31	3	100	-
		5	100	-
		7	100	-
**4**	*M*. *luteus* Pk31	3	100	-
		5	100	-
		7	100	-
**5**	*M*. *luteus* Ob1	3	100	-
		5	100	-
		7	100	-
**6**	*M*. *luteus* WSP45	3	96.3	3.7
		5	86.0	14.0
		7	74.4	25.6
**7**	*A*. *viridans* WSP451	3	100	-
		5	100	-
		7	100	-
**8**	*A*. *sydowii* KGJ10	3	71.2	28.8
		5	45.8	54.2
		7	23.8	76.2

**Fig 1:**
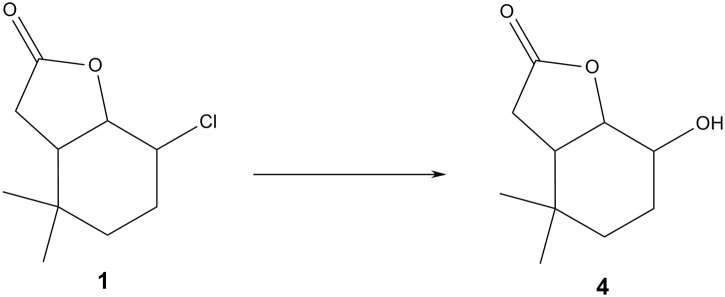
The course of the biotransformation.

It was found that three of the bacterial strains namely *G*. *alcanivorans* Pd25 (entry 1), *M*. *luteus* Pb10 (entry 2) and WSP45 (entry 6) and also the filamentous fungus *A*. *sydowii* KGJ10 (entry 8) were capable of transforming chlorolactone **1**. In all cases biotransformation resulted in the same product—hydroxylactone **4**. The other bacterial strains were not able to convert this substrate into any product. Two further substrates—chlorolactones **2** and **3** –were not transformed by any of the tested microorganisms (Fig 1).

The obtained results were surprising because some strains of *Aspergillus*, especially *A*. *niger* are known for their hydroxylation ability [44–46]. Based on this information we expected to obtain chlorohydroxylactone rather than hydroxylactone.

**Fig 2:**
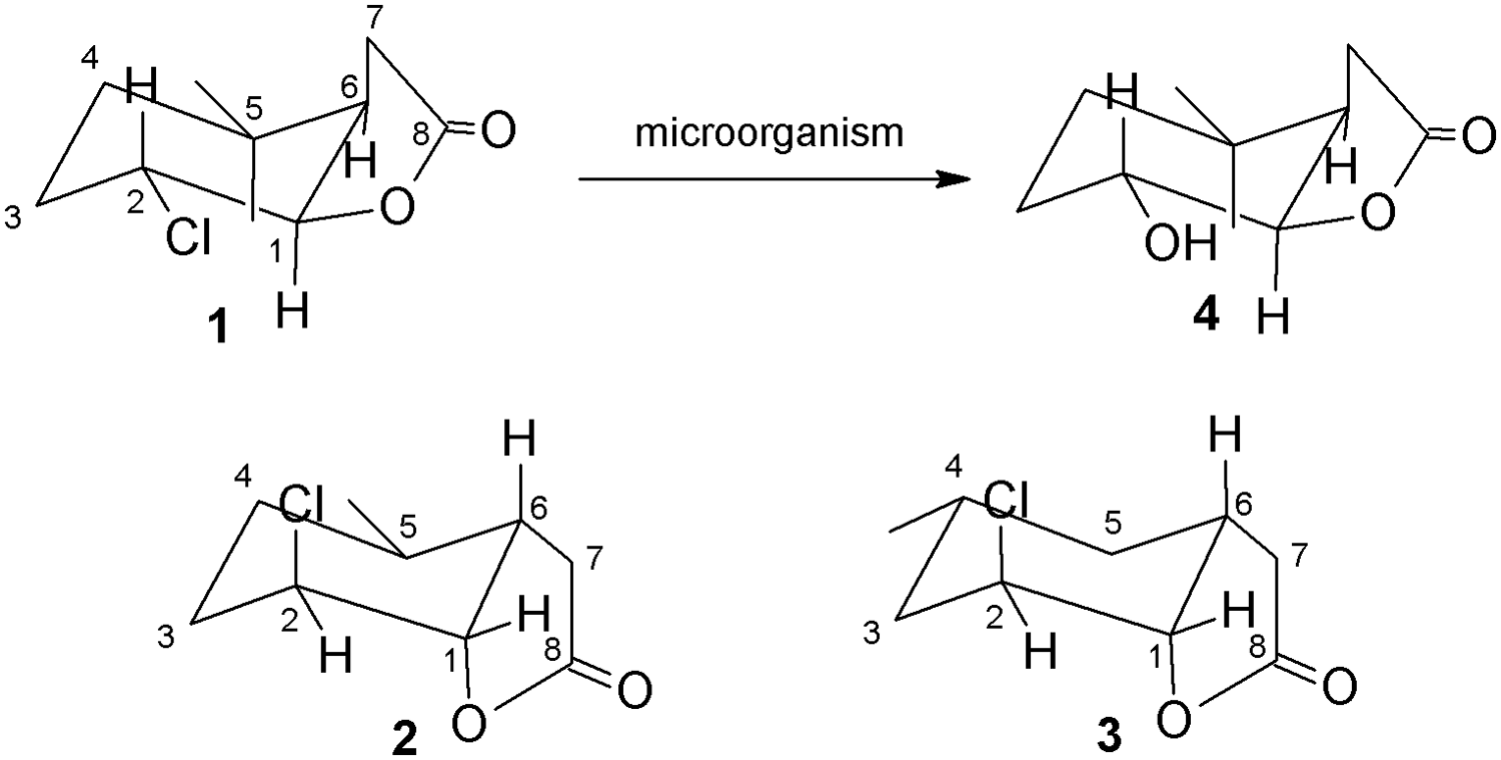
Structures of substrates and product of biotransformation.

The next step was to carry out biotransformation of the substrate **1** at the preparative scale. In the case of the bacterial strains, the degree of conversion of the substrate did not exceed 30%. When the *A*. *sydowii* strain was used as a biocatalyst it was possible to obtain a product with a yield above 76%. Because in all cases the same product was created (according to the GC retention time) the *A*. *sydowii* strain was used to carry out the preparative biotransformation. As a result, hydroxylactone **4** was obtained in a yield of 74.3% according to GC analysis. After isolation and purification of the biotransformation product, 0.0262g (32.1%) of pure hydroxylactone **4** was obtained. Analysis of the NMR spectra (S1–S3 Figs) showed that this was a new compound, different to the hydroxylactone obtained during the earlier biotransformation of chlorolactone **1** [14] (Fig 2).

Analysis of the ^1^H NMR spectra of chlorolactones **1**, **2** and **3** made it possible to prove that their structures were different (Fig 2). The only common feature of these compounds was that cyclohexane ring took the chair conformation. In the case of chlorolactone **1** the signals with large coupling constants coming from protons H-1, H-2 and H-6 demonstrate their axial orientation. This means that at the same time, the chlorine atom located at carbon C-2 was in an equatorial position. In the case of chlorolactone **2** signals coming from protons H-1 and H-2 appear as a narrow singlets, suggesting their diequatorial position. This means therefore that the chlorine atom located at carbon C-2 occupied the axial position. A similar situation exists in the case of chlorolactone **3**. This time the signals derived from protons H-1 and H-2 were multiplets with small coupling constants, which indicates their diequatorial position and the same the axial orientation of the chlorine atom located at carbon C-2. The broad multiplet coming from the proton H-6 also indicated its axial position. Thus, the different structures of the three substrates likely affected the susceptibility of chlorolactones **1**–**3** to biotransformation, which was observed during screening biotransformation. Only chlorolactone **1** was transformed by some of the microorganisms. Additionally, the structure of hydroxylactone **4** obtained during the biotransformation of chlorolactone **1** was very similar to the structure of the substrate. Signals with the large coupling constants derived from protons H-2 and H-1 indicated their axial orientation. This means therefore that the hydroxy group occupied the equatorial position. It turned out that the microorganisms selected for the test had a strong preference for the arrangement the chlorine atom in equatorial position, because they were then capable of exchanging the chlorine atom with a hydroxy group. This was a very interesting result, because during the earlier experiments carried out by our team this type of relationship has not been observed. Moreover, the microorganisms used for the dehalogenation carry out these reactions with a mechanism resembling S_N_2 mechanism. In addition, the hydroxy group was almost always introduced in an equatorial position. There were observed two different possibilities of the product formation. The first was preservation of the conformation of the cyclohexane ring when the halogen atom was in an axial position. The second was a change of conformation of the cyclohexane ring when a halogen occupied an equatorial position. The only exception were halolactones with two methyl groups located at carbon C-5. In this case, the conformation of the cyclohexane ring was not changed, but the hydroxy group was introduced in an axial position [14]. The analogous compound to our substrate **1** (iodine in the equatorial position) was transformed by the strain *Absidia cylindrospora* KCh336. [47]. The formation of the product with OH group in the axial position was observed in this research, what suggested that the reaction was according to the S_N_2 mechanism. In the experiment described here, there was also no change of the conformation of the cyclohexane ring, despite the fact that the substrate chlorine atom was in the equatorial position. Interestingly, the hydroxy group of the product was also introduced in an equatorial position. This means that the reaction mechanism of hydrolytic dehalogenation in this case was different to that normally observed. In addition, compounds with the chlorine atom lying in the axial position were not transformed at all. It can therefore be concluded that the hydroxy group was introduced by a simultaneous elimination-substitution mechanism or S_N_1 mechanism. The first proposed mechanism should result in two products in which the OH group is at C2 or C3. Formation of a product with a hydroxyl group at C3 was not observed at all, what suggests the S_N_1 mechanism. The fact that the resulting product is a racemic mixture (ee = 2.5%, ${\left[a\right]}_{20}^{D}=+7.22$ (c = 0.765g/100mL) supports both hypothesis.

The classification of the strains, that were able to transform chlorolactone **1** was confirmed by DNA analysis.

**Fig 3:**
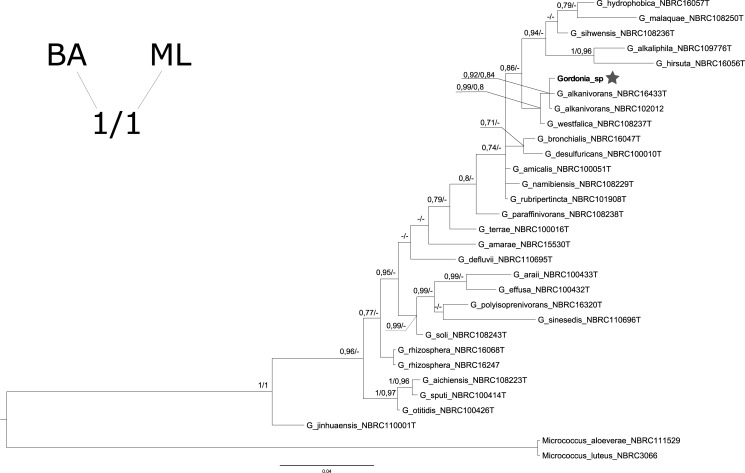
Bayesian phylogenetic tree of 29 *Gordonia* sp. representatives and two *Micrococcus* sp. used for rooting. Numbers along the nodes are the posterior probabilities of the nodes (BA) and the maximum likelihood bootstrap values (ML) (values below 0.7 were not shown (−)). The DNA sequence of *Gordonia* sp. obtained in this study is presented in bold and marked with a star.

Sequencing of the Pb10 sample showed a DNA haplotype (Genbank accession number: KY948067) which presented 100% complementarity to the *M*. *luteus* 16S rRNA gene (FN393778) already present in the Genbank database. Thus, we can conclude that the Pb10 sample was collected from *M*. *luteus*. DNA sequence derived from the Pd25 sample (Genbank accession no: KY948023), was identified as *Gordonia* sp. when we used Blast to examine its systematic identity. To narrow down the taxonomic position we created the phylogenetic tree presented in Fig 3. Both trees (BA and ML) generated in this project had similar topology, with only minor differences between them. *Gordonia* species sequence from our study was grouped on together with two *G*. *alkanivorans* sequences, and the node connecting those sequences had a high probability (Bayesian posterior probability– 0.92 and bootstrap analysis probability– 0.84). Therefore, we can state that analysed Pd25 sample was representative of *G*. *alkanivorans*.

**Fig 4:**
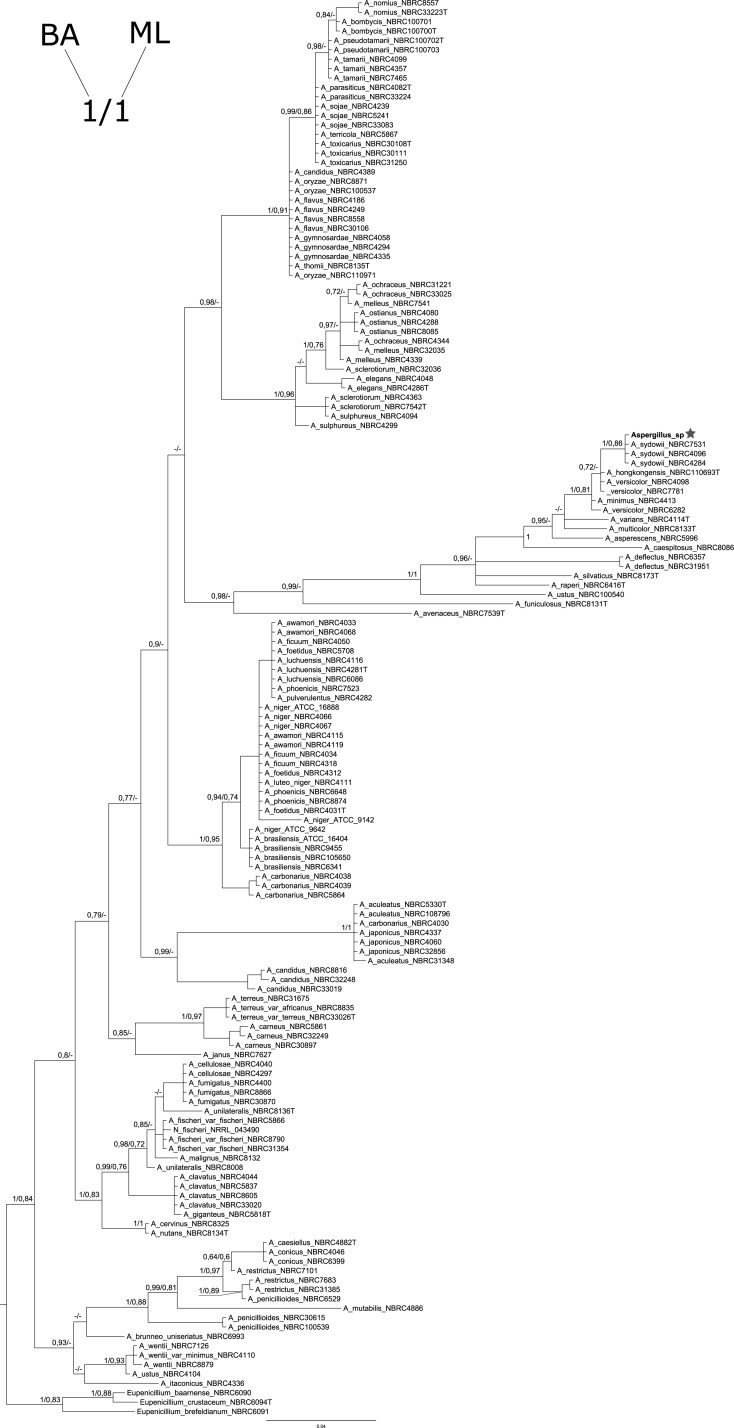
Bayesian phylogenetic tree of 148 *Aspergillus* sp. representatives and three *Eupenicillium* sp. sequences used as an outgroup. Numbers along the nodes are the posterior probabilities of the nodes (BA) and maximum likelihood bootstrap values (ML) (values below 0.7 were not shown (−)). The DNA sequence of the *Aspergillus* sp. obtained in this study is presented in bold and marked with a star.

Analogous results are also shown for the fungal sample. In Blast analysis we were only able to identify the strain as a representative of an *Aspergillus* species (Genbank accession no: KY948068). Once more, to narrow down the results we created a phylogenetic tree, which is presented in Fig 4. Again, both trees (BA and ML) presented almost identical topologies. The *Aspergillus* sample from this study was placed on the tree in a clade containing three representatives of *A*. *sydowii*. The node connecting all those sequences was highly significant as its Bayesian posterior probability was 1 and its bootstrap value was 0.86. Once more, thanks to phylogenetic analysis we were able to identify the exact species of our sample as *A*. *sydowii*.

It is known from the literature that strains of *M*. *luteus* and *A*. *sydowii* have high osmotolerance [48] and have been previously detected in the Bochnia salt mine [49].

During biotransformation, creating a different product than we expected may be a consequence of the genetic diversity present within the genus *Aspergillus* (Fig 4). The *A*. *sydowii* clade presented in the phylogenetic tree (Fig 4) is located in a different part of the tree and thus differs from strains of *Aspergillus* that have previously been used in biotransformations.

## Conclusion

Three bicyclic chlorolactones containing one or two methyl groups in the cyclohexane ring were biotransformed using the new untested bacterial strains and one filamentous fungal strain derived from the salt mine. Most of the microorganisms were able to carry out the hydrolytic dehalogenation only of the substrate with two methyl groups. The equatorial position of the chlorine atom in the compound was decisive for the course of the reaction. The other two substrates, wherein the chlorine atom was in an axial position were not transformed. The biotransformation resulted in a new hydroxylactone. The mechanism of the reaction was different from that previously observed, as the hydroxy group was introduced in an equatorial position without changing the conformation of the molecule.

## Supporting information

S1 Table The list of sequences used for phylogentic analysis of *Gordonia* sp. sample. (DOCX)

S2 Table The list of sequences used for phylogentic analysis of *Aspergillus* sp. sample. (DOCX)

S1 Fig ^1^H NMR (600 MHz, CDCl_3_) spectrum of hydroxylactone 4. (DOCX)

S2 Fig COSY (151 MHz, CDCl_3_) spectrum of hydroxylactone 4. (DOCX)

S3 Fig HMQC (151 MHz, CDCl_3_) spectrum of hydroxylactone 4. (DOCX)

S4 Fig ^13^C NMR (151 MHz, CDCl_3_) spectrum of hydroxylactone 4. (DOCX)

S5 Fig HRMS-ESI spectrum of hydroxylactone 4 (MeOH with 0.1% of formic acid). (DOCX)
