# Supplementary material for: Salt mine microorganisms used for the biotransformation of chlorolactones
Source: PLoS One. 2018 May 17;13(5):e0197384. doi: 10.1371/journal.pone.0197384 (PMC5957361; doi:10.1371/journal.pone.0197384)
Supplement: S1 Table — (DOCX) [file pone.0197384.s001.docx]

**S1 Table.** **The list of sequences used for phylogentic analysis of *Gordonia* sp. sample.**

| **No.** | **Species** | **Database number** |
| --- | --- | --- |
| **1** | G. aichiensis | NBRC108223T |
| **2** | G. alkaliphila | NBRC109776T |
| **3** | G. alkanivorans | NBRC102012 |
| **4** | G. alkanivorans | NBRC16433T |
| **5** | G. amarae | NBRC15530T |
| **6** | G. amicalis | NBRC100051T |
| **7** | G. araii | NBRC100433T |
| **8** | G. bronchialis | NBRC16047T |
| **9** | G. defluvii | NBRC110695T |
| **10** | G. desulfuricans | NBRC100010T |
| **11** | G. effusa | NBRC100432T |
| **12** | G. hirsuta | NBRC16056T |
| **13** | G. hydrophobica | NBRC16057T |
| **14** | G.jinhuaensis | NBRC110001T |
| **15** | G. malaquae | NBRC108250T |
| **16** | G. namibiensis | NBRC108229T |
| **17** | G. otitidis | NBRC100426T |
| **18** | G. paraffinivorans | NBRC108238T |
| **19** | G. paraffinivorans | NBRC108238T |
| **20** | G. polyisoprenivorans | NBRC16320 |
| **21** | G. rhizosphera | NBRC16068T |
| **22** | G. rhizosphera | NBRC16247 |
| **23** | G. rubripertincta | NBRC101908T |
| **24** | G. sihwensis | NBRC108236T |
| **25** | G. sinesedis | NBRC110696T |
| **26** | G. soli | NBRC108243T |
| **27** | G. sputi | NBRC100414T |
| **28** | G. terrae | NBRC100016T |
| **29** | G. westfalica | NBRC108237T |
| **30** | M. aloeverae | NBRC1115 |
| **31** | M. luteus | NBRC3066 |
