# Supplementary material for: Salt mine microorganisms used for the biotransformation of chlorolactones
Source: PLoS One. 2018 May 17;13(5):e0197384. doi: 10.1371/journal.pone.0197384 (PMC5957361; doi:10.1371/journal.pone.0197384)
Supplement: S2 Table — (DOCX) [file pone.0197384.s002.docx]

**S2 Table.** **The list of sequences used for phylogentic analysis of *Aspergillus* sp. sample.**

| **No.** | **Species** | **Database number** |
| --- | --- | --- |
| **1** | A. aculeatus | NBRC108796 |
| **2** | A. aculeatus | NBRC31348 |
| **3** | A. aculeatus | NBRC5330T |
| **4** | A. asperescens | NBRC5996 |
| **5** | A. avenaceus | NBRC7539T |
| **6** | A. awamori | NBRC4033 |
| **7** | A. awamori | NBRC4068 |
| **8** | A. awamori | NBRC4115 |
| **9** | A. awamori | NBRC4119 |
| **10** | A. bombycis | NBRC100700T |
| **11** | A. bombycis | NBRC100701 |
| **12** | A. brasilensis | ATCC16404 |
| **13** | A. brasiliensis | NBRC105650 |
| **14** | A. brasiliensis | NBRC6341 |
| **15** | A. brasiliensis | NBRC9455 |
| **16** | A. brunneo uniseriatus | NBRC6993 |
| **17** | A. caesiellus | NBRC4882T |
| **18** | A. caespitosus | NBRC8086 |
| **19** | A. candidus | NBRC32248 |
| **20** | A. candidus | NBRC33019 |
| **21** | A. candidus | NBRC4389 |
| **22** | A. candidus | NBRC8816 |
| **23** | A. carbonarius | NBRC4030 |
| **24** | A. carbonarius | NBRC4038 |
| **25** | A. carbonarius | NBRC4039 |
| **26** | A. carbonarius | NBRC5864 |
| **27** | A. carneus | NBRC30897 |
| **28** | A. carneus | NBRC32249 |
| **29** | A. carneus | NBRC5861 |
| **30** | A. cellulosae | NBRC4040 |
| **31** | A. cellulosae | NBRC4297 |
| **32** | A. cervinus | NBRC8325 |
| **33** | A. clavatus | NBRC33020 |
| **34** | A. clavatus | NBRC4044 |
| **35** | A. clavatus | NBRC5837 |
| **36** | A. clavatus | NBRC8605 |
| **37** | A. conicus | NBRC4046 |
| **38** | A. conicus | NBRC6399 |
| **39** | A. deflectus | NBRC31951 |
| **40** | A. deflectus | NBRC6357 |
| **41** | A. elegans | NBRC4048 |
| **42** | A. elegans | NBRC4286T |
| **43** | A. ficuum | NBRC4034 |
| **44** | A. ficuum | NBRC4050 |
| **45** | A. ficuum | NBRC4318 |
| **46** | A. fischeri | NRRL43490 |
| **47** | A. fischeri | NBRC31354 |
| **48** | A. fischeri | NBRC5866 |
| **49** | A. fischeri | NBRC8790 |
| **50** | A. flavus | NBRC30106 |
| **51** | A. flavus | NBRC4186 |
| **52** | A. flavus | NBRC4249 |
| **53** | A. flavus | NBRC8558 |
| **54** | A. foetidus | NBRC4031T |
| **55** | A. foetidus | NBRC4312 |
| **56** | A. foetidus | NBRC5708 |
| **57** | A. fumigatus | NBRC30870 |
| **58** | A. fumigatus | NBRC4400 |
| **59** | A. fumigatus | NBRC8866 |
| **60** | A. funiculosus | NBRC8131T |
| **61** | A. giganteus | NBRC5818T |
| **62** | A. gymnosardae | NBRC4058 |
| **63** | A. gymnosardae | NBRC4294 |
| **64** | A. gymnosardae | NBRC4335 |
| **65** | A. hongkongensis | NBRC110693T |
| **66** | A. itaconicus | NBRC4336 |
| **67** | A. janus | NBRC7627 |
| **68** | A. japonicus | NBRC32856 |
| **69** | A. japonicus | NBRC4060 |
| **70** | A.japonicus | NBRC4337 |
| **71** | A. luchuensis | NBRC4116 |
| **72** | A. luchuensis | NBRC4281T |
| **73** | A. luchuensis | NBRC6086 |
| **74** | A. luteo niger | NBRC4111 |
| **75** | A. malignus | NBRC8132 |
| **76** | A. melleus | NBRC32035 |
| **77** | A. melleus | NBRC4339 |
| **78** | A. melleus | NBRC7541 |
| **79** | A. minimus | NBRC4413 |
| **80** | A. multicolor | NBRC8133T |
| **81** | A. mutabilis | NBRC4886 |
| **82** | A. niger | ATCC16888 |
| **83** | A. niger | ATCC9142 |
| **84** | A. niger | ATCC9642 |
| **85** | A. niger | NBRC4066 |
| **86** | A. niger | NBRC4067 |
| **87** | A. nomius | NBRC33223T |
| **88** | A. nomius | NBRC8557 |
| **89** | A. nutans | NBRC8134T |
| **90** | A. ochraceus | NBRC31221 |
| **91** | A. ochraceus | NBRC33025 |
| **92** | A. ochraceus | NBRC4344 |
| **93** | A. oryzae | NBRC100537 |
| **94** | A. oryzae | NBRC110971 |
| **95** | A. oryzae | NBRC8871 |
| **96** | A. ostianus | NBRC4080 |
| **97** | A. ostianus | NBRC4288 |
| **98** | A. ostianus | NBRC8085 |
| **99** | A. parasiticus | NBRC33224 |
| **100** | A. parasiticus | NBRC4082T |
| **101** | A. penicillioides | NBRC100539 |
| **102** | A. penicillioides | NBRC30615 |
| **103** | A. penicillioides | NBRC6529 |
| **104** | A. phoenicis | NBRC6648 |
| **105** | A. phoenicis | NBRC7523 |
| **106** | A. phoenicis | NBRC8874 |
| **107** | A. pseudotamarii | NBRC100702T |
| **108** | A. pseudotamarii | NBRC100703 |
| **109** | A. pulverulentus | NBRC4282 |
| **110** | A. raperi | NBRC6416T |
| **111** | A. restrictus | NBRC31385 |
| **112** | A. restrictus | NBRC7101 |
| **113** | A. restrictus | NBRC7683 |
| **114** | A. sclerotiorum | NBRC32036 |
| **115** | A. sclerotiorum | NBRC4363 |
| **116** | A. sclerotiorum | NBRC7542T |
| **117** | A. silvaticus | NBRC8173T |
| **118** | A. sojae | NBRC33083 |
| **119** | A.sojae | NBRC4239 |
| **120** | A. sojae | NBRC5241 |
| **121** | A. sulphureus | NBRC4094 |
| **122** | A. sulphureus | NBRC4299 |
| **123** | A. sydowii | NBRC4096 |
| **124** | A.sydowii | NBRC4284 |
| **125** | A. sydowii | NBRC7531 |
| **126** | A. tamarii | NBRC4099 |
| **127** | A. tamarii | NBRC4357 |
| **128** | A. tamarii | NBRC7465 |
| **129** | A. terreus | NBRC31675 |
| **130** | A. terreus | NBRC8835 |
| **131** | A. terreus | NBRC3302 |
| **132** | A. terricol | NBRC5867 |
| **133** | A. thomii | NBRC8135T |
| **134** | A. toxicarius | NBRC30108T |
| **135** | A. toxicarius | NBRC30111 |
| **136** | A. toxicarius | NBRC31250 |
| **137** | A. unilateralis | NBRC8008 |
| **138** | A. unilateralis | NBRC8136T |
| **139** | A. ustus | NBRC100540 |
| **140** | A. ustus | NBRC4104 |
| **141** | A. varians | NBRC4114T |
| **142** | A. versicolor | NBRC4098 |
| **143** | A. versicolor | NBRC6282 |
| **144** | A. versicolor | NBRC7781 |
| **145** | A. wentii | NBRC7126 |
| **146** | A. wentii | NBRC8879 |
| **147** | A. wentii | NBRC4110 |
| **148** | Eupenicillium baarnense | NBRC6090 |
| **149** | E. brefeldianum | NBR6094T |
| **150** | E. crustaceum | NBRC6091 |
