# Supplementary figures and images for: Salt mine microorganisms used for the biotransformation of chlorolactones
Source: PLoS One. 2018 May 17;13(5):e0197384. doi: 10.1371/journal.pone.0197384 (PMC5957361; doi:10.1371/journal.pone.0197384)

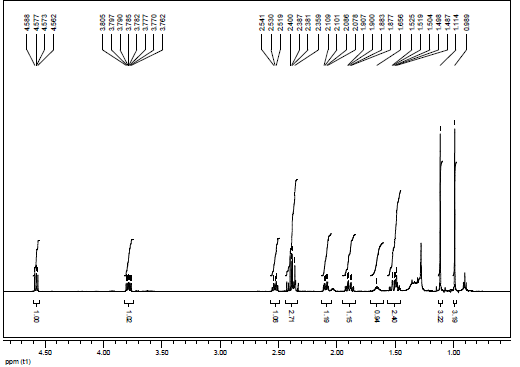

Supplement: S1 Fig — (DOCX) [file pone.0197384.s003.docx]

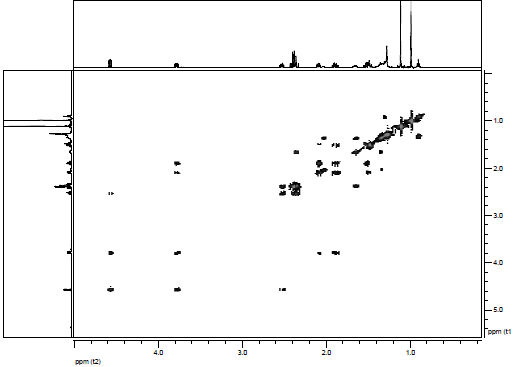

Supplement: S2 Fig — (DOCX) [file pone.0197384.s004.docx]

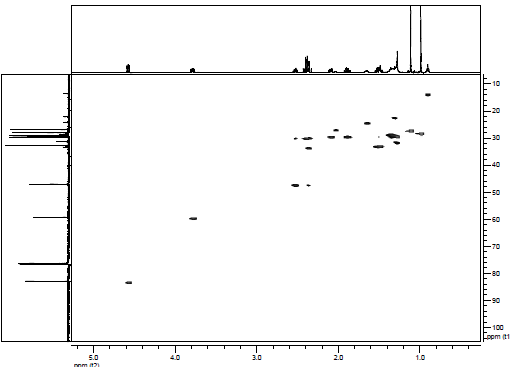

Supplement: S3 Fig — (DOCX) [file pone.0197384.s005.docx]

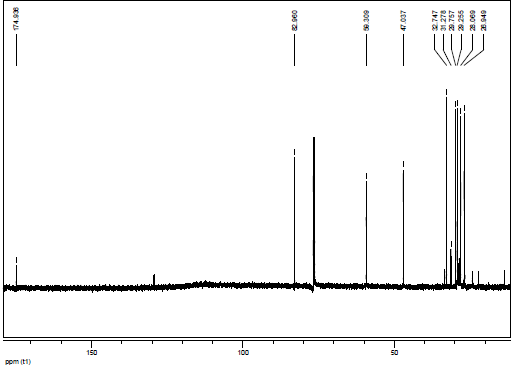

Supplement: S4 Fig — (DOCX) [file pone.0197384.s006.docx]

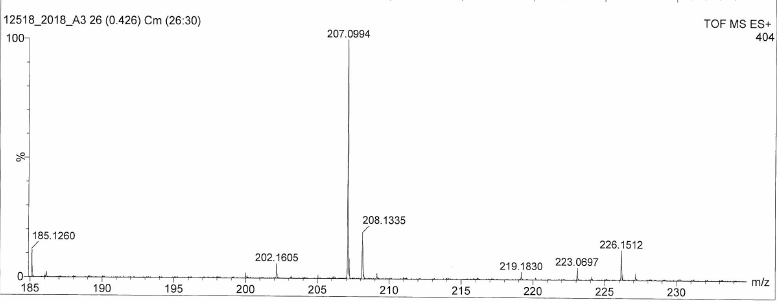

Supplement: S5 Fig — (DOCX) [file pone.0197384.s007.docx]
